# Binding of FAD and tryptophan to the tryptophan 6‐halogenase Thal is negatively coupled

**DOI:** 10.1002/pro.3739

**Published:** 2019-10-21

**Authors:** Ann‐Christin Moritzer, Hartmut H. Niemann

**Affiliations:** ^1^ Department of Chemistry Bielefeld University Bielefeld Germany

**Keywords:** cofactor binding, cofactor regeneration, crystal structure, flavin adenine dinucleotide, flavin‐dependent halogenase, negative cooperativity, substrate binding, tryptophan halogenase

## Abstract

Flavin‐dependent halogenases require reduced flavin adenine dinucleotide (FADH_2_), O_2_, and halide salts to halogenate their substrates. We describe the crystal structures of the tryptophan 6‐halogenase Thal in complex with FAD or with both tryptophan and FAD. If tryptophan and FAD were soaked simultaneously, both ligands showed impaired binding and in some cases only the adenosine monophosphate or the adenosine moiety of FAD was resolved, suggesting that tryptophan binding increases the mobility mainly of the flavin mononucleotide moiety. This confirms a negative cooperativity between the binding of substrate and cofactor that was previously described for other tryptophan halogenases. Binding of substrate to tryptophan halogenases reduces the affinity for the oxidized cofactor FAD presumably to facilitate the regeneration of FADH_2_ by flavin reductases.

## 1 INTRODUCTION

Flavin‐dependent halogenases (FDHs) mediate the regioselective halogenation of a variety of natural products including antibiotics and anticancer compounds.^1^, ^2^ To halogenate their substrates, FDHs require reduced flavin adenine dinucleotide (FADH_2_), molecular oxygen, and halide salts (usually Cl^−^ or Br^−^). The halide ion is bound between the protein and the isoalloxazine ring of the cofactor.^3^ In the first reaction step, FAD(C4a)‐OOH is formed.^4^ The flavin hydroperoxide then oxidizes the halide to hypohalous acid, which is assumed to move through the protein toward the ε‐amino group of a catalytically essential lysine residue.^3^ Kinetic analysis established that substrate chlorination occurs after completion of flavin redox reactions and that the substrate does not participate in reactions leading from FADH_2_ to FAD(C4a)‐oxygenated species to FAD^4^ (Figure S1). In the absence of substrate, a long‐lived chlorinating intermediate is formed, which was proposed to be a lysine chloramine generated by the reaction of HOCl with the active site lysine of the enzyme.^5^ Regioselectivity is achieved by positioning the substrate relative to the ε‐amino group of the catalytic lysine.^6^ Depending on whether its C5, C6, or C7 atom is positioned most closely to this lysine, the substrate tryptophan is halogenated in position 5, 6, or 7 by various tryptophan halogenases. So far, crystal structures of one tryptophan 5‐halogenase (PyrH^6^), two tryptophan 7‐halogenases (PrnA^3^ and RebH^5^, ^7^), and several tryptophan 6‐halogenases (SttH,^8^ Th‐Hal,^9^ Thal,^10^ and Tar14^11^) have been described. Comparison of apo, FAD‐bound and l‐Trp‐bound structures reveals two flexible loops that undergo conformational changes upon binding of substrate or cofactor. When l‐Trp is bound in the active site, it is covered by a substrate‐binding loop that is disordered in structures without substrate. Binding of FAD usually results in a closed conformation of the FAD loop (e.g., in PrnA, PyrH, SttH, and Tar14), while in apo structures either it adopts an open conformation (e.g., in Thal) or it is not resolved (e.g., in PyrH and BrvH^12^).

To close the reaction cycle, FADH_2_ needs to be regenerated. This is achieved by separate NAD(P)H‐dependent flavin reductases.^13^ Often a flavin reductase is present in a biosynthetic gene cluster along with an FDH, forming a two‐component system, for example, RebH and RebF.^14^, ^15^ Rather little is known about the mechanistic details of FAD reduction. For example, it is not completely clear whether FAD can be reduced while still bound to the FDH or whether it has to dissociate from the halogenase. This may be protein‐specific as the FAD affinity appears to vary considerably as inferred from cofactor loading upon purification of overexpressed enzymes.^16^

For RebH, a weaker FAD binding was observed in crystals concomitantly soaked with FAD and l‐Trp, and a negative coupling between cofactor and substrate binding was proposed.^5^, ^7^ We observed a similar phenomenon in Thal (also known as ThdH), a tryptophan 6‐halogenase from *Streptomyces albogriseolus* involved in the biosynthesis of thienodolin.^17^, ^18^ We previously reported the structures of Thal in the apo and Trp‐bound forms.^10^ During our attempt to obtain the structures of Thal bound to FAD and in complex with both FAD and l‐Trp, we found that binding of l‐Trp and FAD is negatively coupled as well. Here, we describe these structures of Thal soaked with FAD and with FAD and l‐Trp.

## 2 RESULTS

### 2.1 Structure determination

**Figure 1:**
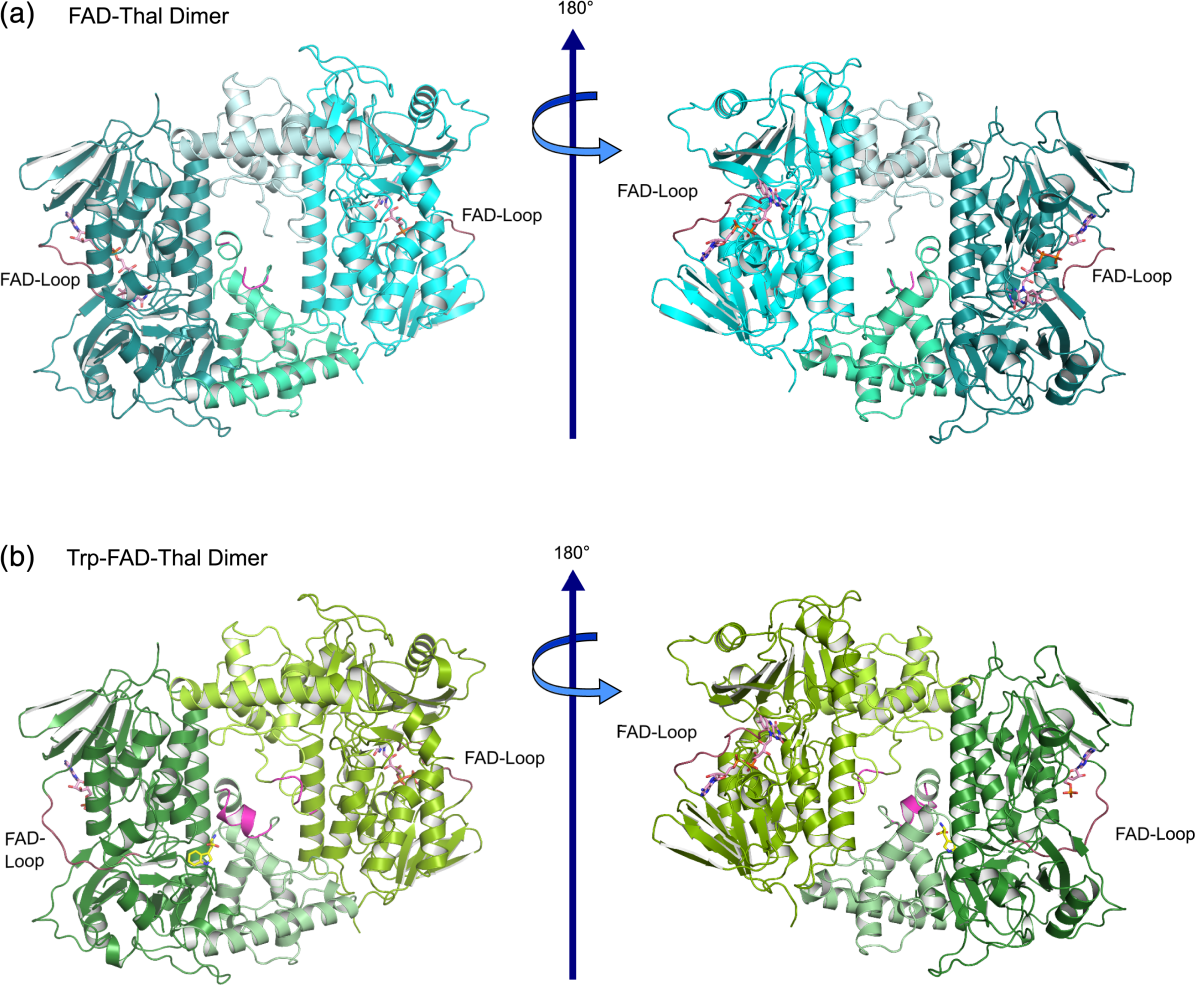
Thal dimer in complex with cofactor or cofactor and substrate. (a) Structure of FAD‐Thal. The box and pyramid subdomains are shown in deep teal and green cyan, as well as cyan and pale cyan, respectively. (b) Structure of Trp‐FAD‐Thal. The box and pyramid subdomains are shown in forest and pale green, as well as split pea green and limon, respectively. The FAD loop that undergoes a conformational change upon FAD binding is highlighted in dark red. The substrate‐binding loop is shown in magenta. The substrate l‐Trp is shown as a stick model with yellow carbon atoms, FAD and AMP are shown with carbons in pink. An enlarged view of the Trp‐FAD‐Thal dimer is provided as Figure S2

Because our attempts to cocrystallize Thal with FAD failed, we soaked crystals of apo‐Thal with FAD and NaCl or with a combination of l‐Trp, FAD, and NaCl resulting in the FAD‐Thal and Trp‐FAD‐Thal structures, respectively (Table 1). As described previously,^10^ Thal crystallized as a dimer in the asymmetric unit with the substrate‐binding loop located at the dimer interface and the FAD loop on the outside (Figure 1 and Figure S2).

**Table 1:** X‐ray data collection and refinement statistics. Values in parentheses are for the highest resolution shell

	FAD‐Thal	Trp‐FAD‐Thal
PDB ID	6SLS	6SLT
Space group	*P*6_4_	*P*6_4_
Unit‐cell constants		
*a = b, c* (Å)	138.6, 142.1	138.3, 142.0
**Data collection statistics**		
Wavelength (Å)	0.97630	1.000
Resolution range (Å)	50.0–2.32 (2.37–2.32)	50.0–2.70 (2.80–2.70)
No. of reflections (measured/unique)	1,391,920/66,881	875,709/42,328
Completeness (%)	100 (100)	100 (100)
*R*_meas_ (%)	6.4 (194.4)	18.1 (178.5)
*R*_pim_ (%)	1.4 (41.9)	4.0 (39.3)
Redundancy	20.8 (21.4)	20.7 (20.4)
Mean *I/σ(I)*	28.5 (1.8)	14.6 (2.1)
CC ½ (%)	100 (81.2)	99.8 (77.5)
Wilson *B* factor (Å^2^)	72	70
**Refinement and model statistics**		
Resolution range	49.64–2.32 (2.38–2.32)	49.58–2.70 (2.77–2.70)
No. of reflections (work/test)	63,482 (4673)/3,359 (254)	40,099 (2963)/2,195 (167)
*R*_work_ (%)	18.0 (29.7)	18.4 (27.8)
*R*_free_ (%)	22.4 (33.1)	24.1 (35.4)
r.m.s.d. bonds (Å)	0.008	0.009
r.m.s.d. angles (°)	1.507	1.588
No. of atoms	8,585	8,621
Protein	8,308	8,404
Ligands	150	142
Solvent	127	75
Average *B* factor (Å^2^)	77	75
Protein	77	75
Ligands	86	90
Solvent	67	56
Ramachandran		
Favored (%)	95.55	93.70
Allowed (%)	4.36	6.02
Outliers (%)	0.10	0.29

### 2.2 Binding of FAD to Thal results in closing of the FAD loop

**Figure 2:**
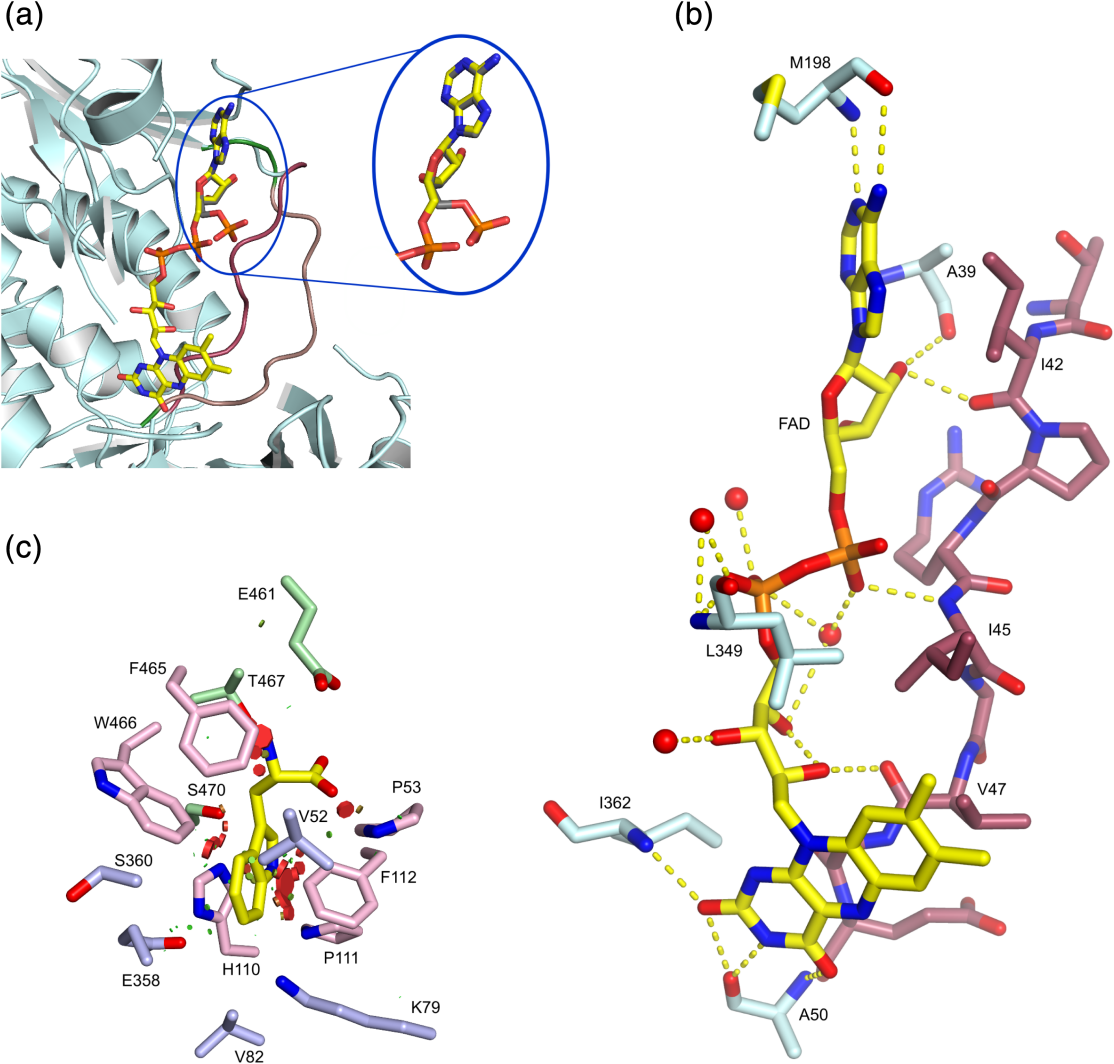
Conformational change of the FAD loop and details of substrate‐binding site. (a) Overlay of the FAD‐binding site of FAD‐Thal (pale cyan and dark red) and the FAD loop of chain A of Trp‐FAD‐Thal (salmon). The cofactor FAD is shown with carbons in yellow and AMP with carbons in grey. The FAD loop is highlighted in dark red in the closed state and in salmon in the open state. In chain A of the Trp‐FAD‐Thal structure, the phosphate of AMP points out of the FAD‐binding site. Several residues are hidden for a better view of the FAD and AMP. (b) Hydrogen bonds between FAD (yellow) and Thal (pale cyan and dark red) or protein‐bound waters are shown as dotted yellow lines. (c) Steric conflicts upon placing l‐Trp from Trp‐Thal in FAD‐Thal. l‐Trp from Trp‐Thal chain A (PDB ID 6H44) was placed in FAD‐Thal chain A. Minor clashes are indicated as small green hexagons, more severe clashes are shown as bigger red hexagons. Clashes mainly occur between the indole moiety of l‐Trp and the side chains of the conserved residues Pro53, Phe112, Phe465, and Trp466. Conserved residues Tyr 454 and Tyr455 are not shown because the substrate‐binding loop is disordered. Coloring of the active site residues according to previously published work^10^

The FAD‐Thal structure contains FAD with an occupancy of 0.9 in both chains (Figure S3). Compared to apo‐Thal (PDB ID 6H43) and Trp‐Thal (PDB ID 6H44), the conformation of the FAD loop changes to the closed conformation (Figures 1a and 2a,b). Upon switching from the open to closed state, Pro40 undergoes a peptide flip and Glu49, the other flanking residue, flips its side chain by almost 180° as in PyrH.^6^ Conformational switching of the FAD loop probably also occurs in the other FDHs, although two conformations have only been observed in PyrH.^6^

To address the potential crystal‐to‐crystal variations of FAD loading and the conformation of flexible loops, we determined the structures of four other Thal crystals soaked with FAD and chloride at resolutions of 2.5–2.8 Å. We did not fully refine or deposit these additional structures, but could reliably extract the following features. In all structures both active sites are empty, FAD binds to both monomers with high occupancy and the FAD loop is closed. The substrate‐binding loop is defined in chain A of three crystals but disordered in the remaining Thal protomers.

To distinguish between a chloride ion with partial occupancy and water in the canonical halide‐binding site formed by the backbone NH groups of Ser360 and Gly361, we collected long‐wavelength data sets (*λ* = 2.1015 Å) for three crystals with an anomalous redundancy of 9.7 each. Anomalous difference density is visible for sulfur atoms and the phosphates in FAD in all three crystals. Only three of the six Thal protomers have weak anomalous difference densities in the canonical halide‐binding site, but no such density is present in the crystal that we fully refined. Therefore, water A763/B741 occupies the catalytic halide‐binding site in the published FAD‐Thal structure (PDB ID 6SLS).

There are structural differences between FAD‐Thal and Trp‐Thal outside the FAD loop and the substrate‐binding loop. Gly13 undergoes a peptide flip so that in FAD‐Thal, the NH of Gly14 rather than the O of Gly13 points toward the FAD phosphates. Val52 and Pro53 move into the tryptophan‐binding site by 0.6 Å–1.1 Å (Figure 2c). These amino acids could be viewed as the continuation of the FAD loop. Thus, subtle structural changes in the tryptophan‐binding site might be directly coupled to the binding of FAD. In PyrH, a similar, although more pronounced, narrowing of the tryptophan‐binding site occurs upon FAD binding. Naismith and coworkers suggested that residues at and following the C‐terminal end of the FAD loop act as a conduit for information transfer between the cofactor‐binding site and the substrate‐binding site.^6^ Further differences between FAD‐Thal and Trp‐Thal may be functionally important. The side chains of Ser360 and Tyr366 adopt other rotamers. In FAD‐Thal, Tyr366 forms a hydrogen bond to the side chain of Asn54, which also interacts with the carboxylate of Glu461. Because Glu461 forms a salt bridge with the α‐amino group of l‐Trp, the residues Tyr366, Asn54, and Glu461 might form another path for coupling the FAD‐binding site and the substrate‐binding site.

### 2.3 Simultaneous soaking of FAD and l‐Trp impairs the binding of cofactor and substrate

By soaking with FAD, NaCl, and l‐Trp, we did not achieve simultaneous high occupancy for FAD and l‐Trp in both chains (Figure 1b and Figure S4). Chain A of Trp‐FAD‐Thal has a well‐defined l‐Trp (occupancy of 0.95), but only the adenosine monophosphate (AMP) moiety of FAD is visible in the electron density (occupancy of 0.96). Reduced FAD binding is not due to crystal contacts because Trp‐FAD‐Thal crystals are isomorphous to FAD‐Thal crystals, which show binding of the complete FAD. The FAD loop is not involved in any crystal contacts (Figure S5). In chain B, the complete FAD is defined (occupancy = 0.93), but there is no clear electron density for l‐Trp. The substrate‐binding loop is ordered in chain A, while it is poorly defined in chain B and, therefore, could not be modeled between residues 454 and 456 (Figure 1b and Figure S2). The FAD loop is closed in chain B, but remains open in chain A despite the clear density for the AMP moiety. This can be rationalized because the adenosine moiety mostly interacts with a nonflexible region (residues 37–40). The phosphate of the AMP moiety does not occupy the same position as in FAD, but points toward the protein surface instead of the binding pocket for the FMN moiety (Figure 2a).

To address potential crystal‐to‐crystal variation, we determined the structures of additional crystals. We did not fully refine or deposit these additional structures, but could reliably extract the features described below. Four additional Thal crystals cocrystallized or soaked with 5 mM l‐Trp (resolution of 2.6 Å–3.0 Å) show a clear difference density for l‐Trp in both chains. We also analyzed four additional Thal crystals soaked with l‐Trp, FAD, and NaCl at resolutions of 2.6–3.1 Å. One crystal shows essentially the same features as described above, except that in chain A only the adenine instead of the AMP moiety of FAD is well defined. In one crystal, l‐Trp and adenosine are visible in both chains. In the last two crystals, l‐Trp is present in both chains, there is no convincing density for any part of FAD in chain A and FAD with extremely high B‐factors could be placed in chain B. To summarize, when tryptophan is bound, mostly only the AMP or adenosine moiety or a poorly bound FAD with high B‐factors was observed.

In the published Trp‐FAD‐Thal structure, the water molecule B713 occupies the possible chloride position and has a lower B‐factor than its surroundings. To distinguish between water and a chloride ion with partial occupancy, we collected long‐wavelength data sets (*λ* = 2.0664 Å) for three Trp‐FAD‐Thal crystals, including the crystal in Table 1, with an anomalous redundancy of about 10.2 each. Anomalous difference density is visible for sulfur atoms and some, but not all, phosphates in FAD or AMP. As there is no anomalous difference density in the canonical halide‐binding site in all three crystals, we only modeled water in the published structure.

## 3 DISCUSSION

Our structures clearly hint to negatively cooperative binding of l‐Trp and FAD. Since the structural changes are minor except for the two flexible loops, it is hard to identify the structural basis of communication between the substrate‐binding site and the FAD‐binding site. The movement of Val52 and Pro53 into the active site when FAD is bound could lead to clashes of Val52 and Pro53 with l‐Trp (Figure 2c). In reverse, binding of l‐Trp could push out Val52 and Pro53. As a result, the FAD loop would be more likely to adopt the open conformation when l‐Trp is bound, thereby promoting the release of the FAD or at least its FMN moiety. The side chain movement of Tyr366 might represent a second way to couple the FAD and the tryptophan‐binding sites via Asn54 and Glu461.

To understand whether negative coupling of substrate and cofactor binding is a more general feature of FDHs, we analyzed all FDHs for which structures with both cofactor and substrate are available. These are the three tryptophan halogenases PrnA,^3^, ^19^, ^20^ RebH,^5^, ^7^ and PyrH,^6^ and the enzymes PltM in complex with phloroglucinol^16^ and MalA′ bound to premalbrancheamide.^21^

MalA′ and PrnA structures provide no hints to negative cooperativity. All structures have one chain per asymmetric unit, and both ligands are bound at occupancies of 1.^3^, ^19^, ^20^, ^21^ Nevertheless, Zhu et al. mention in their paper about PyrH that “Similar disturbances to the flavin site were observed in […] PrnA structures when tryptophan was bound.”^6^

PltM structures show negative coupling between FAD and substrate binding. In the presence of the substrate phloroglucinol, FAD is missing from two of four chains in the asymmetric unit and only portions of FAD are resolved in unusual binding poses in the other two chains.^16^ Although in Thal the AMP moiety of FAD is better resolved, in phloroglucinol‐bound PltM there is no electron density for the ADP part, which is assumed to be flexible. Tsodikov and coworkers suggested that the unusual location of the isoalloxazine moiety may represent a mechanistically important state, in which it can undergo redox chemistry while being sufficiently shielded from solvent.^16^

Similarity is highest between Thal and RebH and PyrH. For PyrH, three structures are available with four chains per asymmetric unit each.^6^ In the absence of substrate, all four chains bind FAD and chloride. In the absence of FAD, all four chains bind tryptophan. In the presence of both FAD and l‐Trp, FAD was modeled in all chains, but l‐Trp was modeled only in chain B. For RebH, seven structures with two chains per asymmetric unit were published, all of them being isomorphous.^5^, ^7^ In FAD‐bound RebH without substrate, FAD was modeled with an occupancy of 1 in both chains. The structure of Trp‐bound, cofactor‐free RebH was obtained by soaking with saturated l‐Trp and 5 mM FAD. The FAD is clearly present in this structure, but was not modeled due to disorder.^5^ In the structure with bound FAD and tryptophan, Bitto et al. modeled FAD with an occupancy of 0.8 in chain A and only the adenosine moiety of FAD with an occupancy of 0.8 in chain B.^7^ Thus, very similar to Thal, binding of tryptophan to RebH destabilizes binding of the FMN moiety in at least one chain of the RebH dimer.

The functional implications of these observations still need to be explored experimentally. Nevertheless, one can put forward a plausible model based on previously published data. Yeh et al. showed for RebH that the formation of hypochlorite, which is coupled to the oxidation of FADH_2_ to FAD, can proceed in the absence of the substrate Trp.^4^ It would make sense that the subsequent binding of tryptophan destabilizes binding of the oxidized cofactor FAD to generate an empty cofactor‐binding site ready to accept reduced FADH_2_ for the next reaction cycle. In the related two‐component flavin‐dependent monooxygenases, results from many systems indicated that flavin transfer between reductase and monooxygenase can be achieved by free diffusion.^22^ Alternatively, the structures could suggest that FAD does not exchange completely but stays bound to Thal or RebH via its AMP or adenosine moiety. We interpret the AMP density in our structure as a result of high mobility in the FMN part of FAD rather than hydrolysis of the cofactor. The FMN moiety would thus leave the halogenase and could be reduced by a NAD(P)H‐dependent flavin reductase. At least some of the heterologous flavin reductases commonly used to provide FADH_2_ for FDHs in in vitro halogenation assays like SsuE^13^, ^16^ or Fre^20^ from *E. coli* accept FMN or even prefer FMN over FAD.^23^, ^24^ The flavin nucleotide‐bound crystal structures of SsuE^25^ and Th‐Fre, a flavin reductase from *Bacillus subtilis*,^9^ contain FMN. Thus, it may not be completely unreasonable that the adenosine or the AMP moiety remain bound to the halogenase while the flipped‐out FMN moiety can be accessed by a flavin reductase.

## 4 MATERIALS AND METHODS

### 4.1 Protein expression, purification, and crystallization

Thal was expressed and purified as described.^10^ Purified Thal was crystallized using the sitting drop vapor‐diffusion method at 20 °C with a drop ratio of 2:1 of protein solution (∼15 mg·mL^−1^) and reservoir solution (0.1 M bicine pH 8.4, 1.6 M K_2_HPO_4_/KH_2_PO_4_ or 0.1 M HEPES pH 7.8, 1.3 M K_2_HPO_4_/NaH_2_PO_4_). Crystals appeared as hexagonal prisms within 7 days. FAD‐Thal crystals were obtained by soaking Thal crystals in reservoir solution additionally containing 12.4 mM FAD and 10 mM NaCl for 45 min. To obtain an l‐Trp‐FAD bound structure, Thal crystals were soaked in reservoir solution additionally containing 5 mM l‐Trp, 5 mM FAD, and 200 mM NaCl for 85 min. For cryoprotection, the crystals were transferred to reservoir solution supplemented with ∼25 % glycerol and ligands before flash cooling in liquid nitrogen.

### 4.2 Data collection, structure determination, and refinement

All data were collected at a temperature of 100 K at beamline P13 operated by EMBL Hamburg at the PETRA III storage ring at DESY, Hamburg, Germany.^26^ The data sets were processed with XDS and scaled with XSCALE.^27^ Both structures were determined by molecular replacement using the program Phaser^28^ and the apo‐Thal structure (PDB ID 6H43) as search model. The structures were improved by modeling in COOT^29^ and restrained refinement in Refmac5^30^ using noncrystallographic symmetry restraints. Structures were built to near completion before placing ligands into difference density. The occupancy of all ligands was refined. The figures of the final structures were generated using PyMOL. The coordinates and structure factors of the FAD‐Thal and Trp‐FAD‐Thal structures were deposited in the Protein Data Bank with accession codes 6SLS and 6SLT, respectively.

## CONFLICT OF INTEREST

The authors declare no potential conflict of interest.

## Supporting information


**Data S1**: The supplementary material contains a scheme of the reaction cycle (Figure S1), an enlarged view of the Trp‐FAD‐Thal dimer (Figure S2), electron density of bound ligands (Figures S3 and S4) and a view of the crystal packing (Figure S5).
