# Supplementary material for: Binding of FAD and tryptophan to the tryptophan 6‐halogenase Thal is negatively coupled
Source: Protein Sci. 2019 Oct 21;28(12):2112–8. doi: 10.1002/pro.3739 (PMC6863734; doi:10.1002/pro.3739)
Supplement: Supplementary file 1 — Data S1: The supplementary material contains a scheme of the reaction cycle (Figure S1), an enlarged view of the Trp‐FAD‐Thal dimer (Figure S2), electron density of bound ligands (Figures S3 and S4) and a view of the crystal packing (Figure S5). [file PRO-28-2112-s001.pdf]

Supplementary Material

Binding of FAD and tryptophan to the tryptophan 6-halogenase Thal are negatively coupled

Ann-Christin Moritzer and Hartmut H. Niemann

Structural Biochemistry, Department of Chemistry, Bielefeld University,
Universitätsstraße 25, D-33615 Bielefeld, Germany

Binding of FAD and tryptophan to the tryptophan 6-halogenase Thal are negatively coupled

Ann-Christin Moritzer and Hartmut H. Niemann

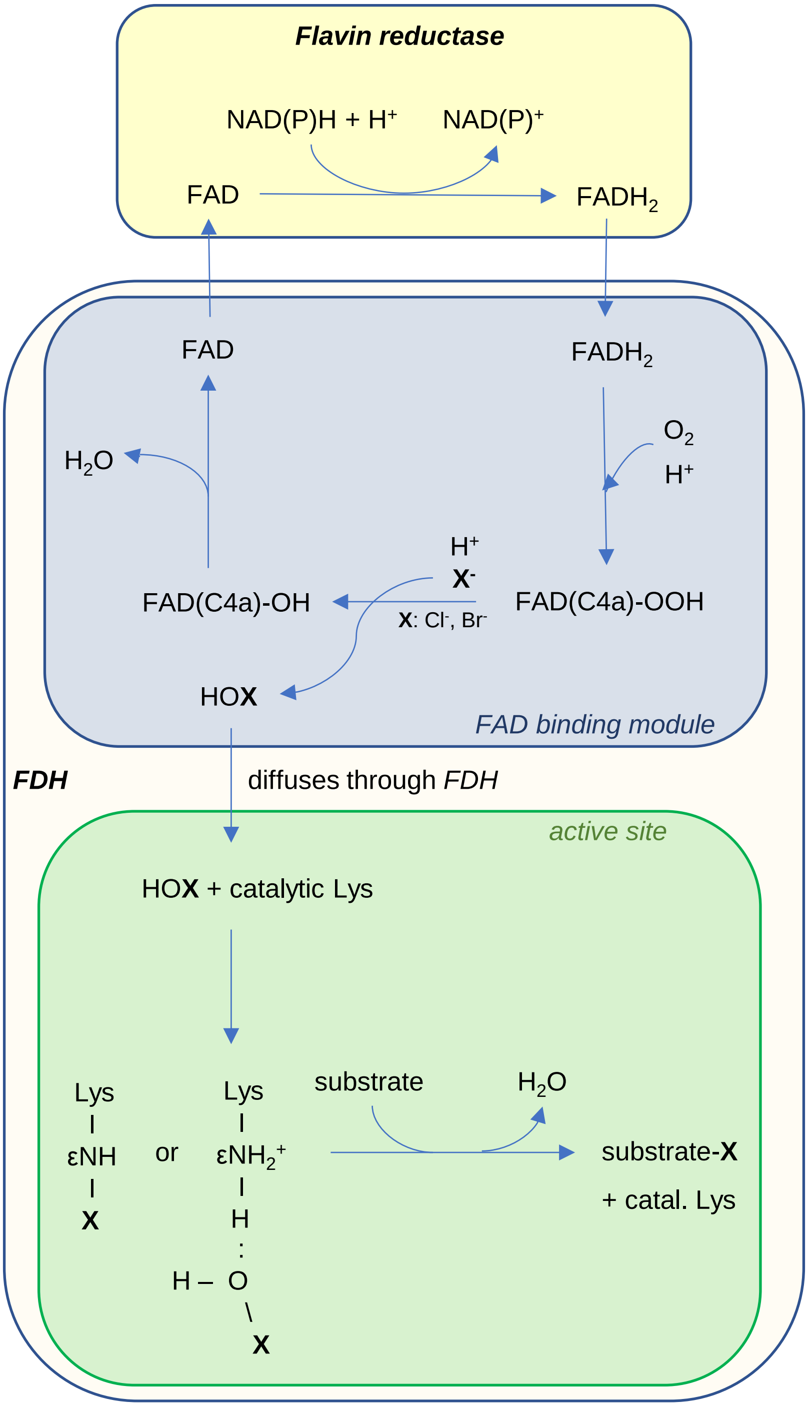

Supplementary Figure S1. Schematic reaction mechanism of two-component flavin-dependent halogenases (FDHs). A separate flavin reductase (yellow) reduces FAD to $FADH_2$. Once bound to the FDH (beige) within the FAD binding module (blue), $FADH_2$ reacts with molecular oxygen to a flavin hydroperoxide. The FAD(C4a)-OOH then reacts with a halide anion forming hypohalous acid (HOX) and FAD(C4a)-OH. The hypohalous acid diffuses through the enzyme towards the active site (green), where halogenation of the substrate takes place. Whether the conserved catalytic lysine forms a hydrogen bond with HOX to orient and activate it or whether it forms a covalent lysine haloamine (e.g. lysine chloramine) is still under debate.

Binding of FAD and tryptophan to the tryptophan 6-halogenase Thal are negatively coupled

Ann-Christin Moritzer and Hartmut H. Niemann

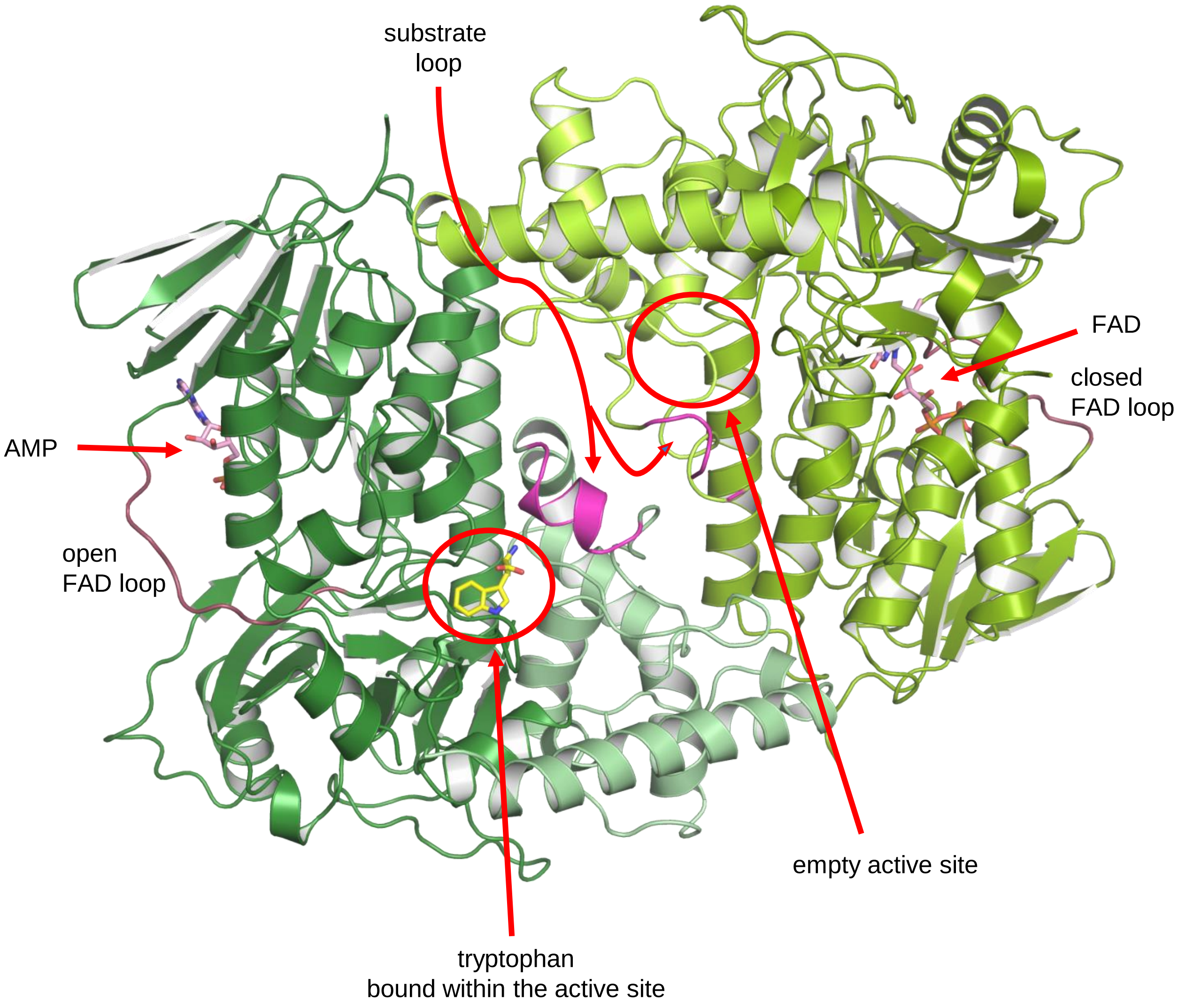

Supplementary Figure S2. Trp-FAD-Thal dimer with regions of interest labeled. In chain A (dark green) the substrate tryptophan (yellow) is bound within the active site, the substrate loop (magenta) is ordered forming a short helix, AMP (pink) is bound within the FAD binding module and the FAD loop (darkpink) adopts an open conformation. In chain B (green) the active site is empty, the substrate loop is not resolved (start and end of the disordered region are shown in magenta), FAD (pink) is bound within the FAD binding module and, therefore, the FAD loop adopts a closed conformation.

Binding of FAD and tryptophan to the tryptophan 6-halogenase Thal are negatively coupled

Ann-Christin Moritzer and Hartmut H. Niemann

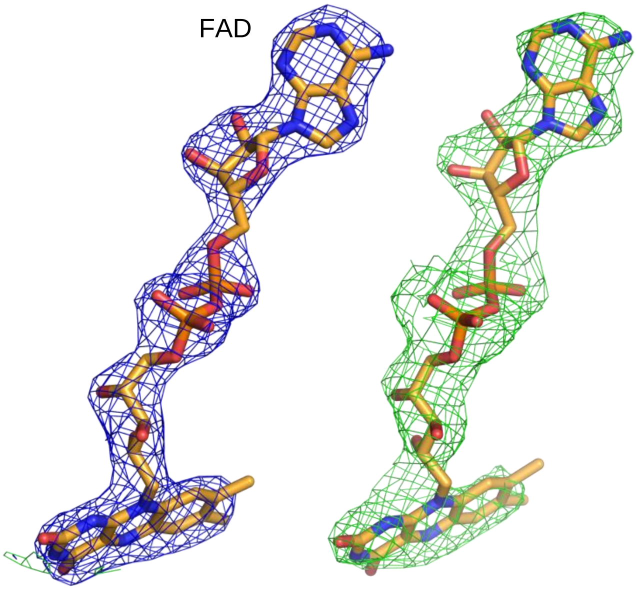

Supplementary Figure S3. Electron density of the cofactor FAD in the FAD-Thal structure. Fo-Fc electron density before building FAD contoured at +3 σ (green) and the final 2Fo-Fc electron density contoured at 1 σ (blue).

Binding of FAD and tryptophan to the tryptophan 6-halogenase Thal are negatively coupled

Ann-Christin Moritzer and Hartmut H. Niemann

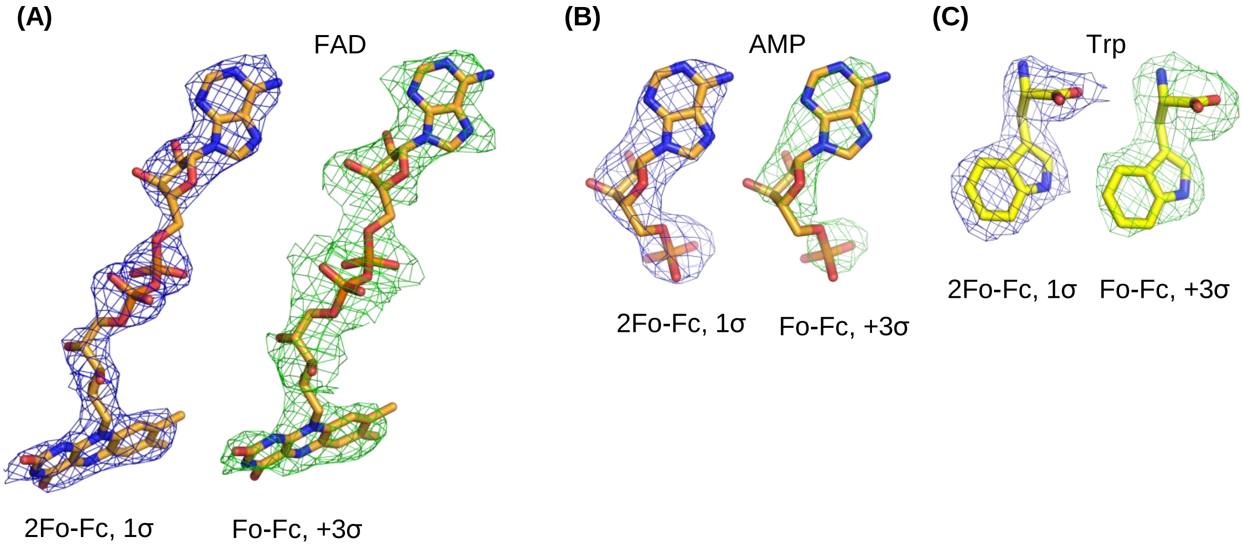

Supplementary Figure S4. Electron density of the ligands in the Trp-FAD-Thal structure. **(A)** Fo-Fc electron density before building the cofactor FAD contoured at +3 σ (green) and the final 2Fo-Fc electron density contoured at 1 σ (blue). **(B)** Fo-Fc electron density before building AMP contoured at +3 σ (green) and the final 2Fo-Fc electron density contoured at 1 σ (blue). **(C)** Fo-Fc electron density before building tryptophan contoured at +3 σ (green) and the final 2Fo-Fc electron density contoured at 1 σ (blue).

Binding of FAD and tryptophan to the tryptophan 6-halogenase Thal are negatively coupled

Ann-Christin Moritzer and Hartmut H. Niemann

(A) (B) (C)

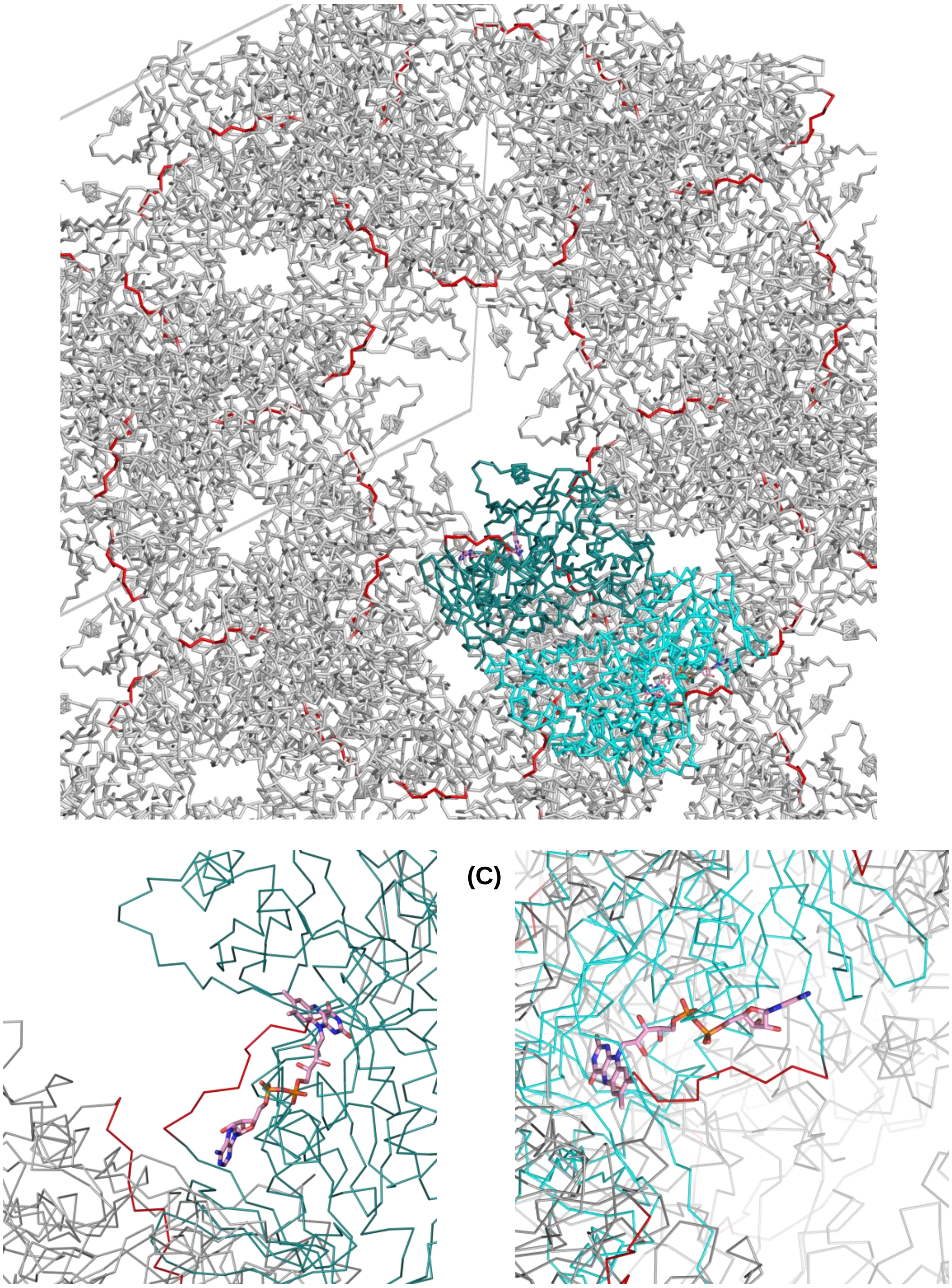

Supplementary Figure S5. Crystal packing of FAD-Thal. **(A)** View along the c axis. The FAD-Thal dimer is colored in cyan and deepteal , symmetry mates are shown in gray and the FAD loops in red. Both FAD loops show no crystal contacts to symmetry mates. **(B, C)** The FAD loop is colored in red. Bound FAD is shown as stick model in pink. **(B)** Zoom in on the FAD loop of chain B of the FAD-Thal dimer. **(C)** Zoom in on the FAD loop of chain A of the FAD-Thal dimer.
